# ASSOCIATED FACTORS WITH THE ISOLATED AND SIMULTANEOUS PRESENCE OF OVERWEIGHT AND ABDOMINAL OBESITY IN ADOLESCENTS

**DOI:** 10.1590/1984-0462/2020/38/2018332

**Published:** 2020-05-08

**Authors:** Tiago Rodrigues de Lima, Mikael Seabra Moraes, Joaquim Huaina Cintra Andrade, Joni Márcio de Farias, Diego Augusto Santos Silva

**Affiliations:** a Universidade Federal de Santa Catarina, Florianópolis, SC, Brazil.; b Universidade do Extremo Sul Catarinense, Criciúma, SC, Brazil.

**Keywords:** Epidemiology, Adolescent health, Risk factors, Public health, Cross-sectional studies, Epidemiologia, Saúde do adolescente, Fatores de risco, Saúde pública, Estudos transversais

## Abstract

**Objective:**

To analyze the sociodemographic and lifestyle factors associated with excessive weight (EW), abdominal obesity (AO) and the simultaneous presence of EW and AO in adolescents from Southern Brazil.

**Methods:**

Cross-sectional study with 583 adolescents (11 to 17 years old) of Criciúma, Santa Catarina, Brazil. EW was assessed by body mass index (BMI) and AO by waist circumference (WC). The independent variables analyzed were gender, age, maternal schooling, balanced diet, physical activity, cigarette use, excessive alcohol use and screen time. Binary logistic regression was used to estimate *Odds Ratios* (OR) and 95% confidence intervals (95%CI).

**Results:**

Boys had 58% higher likelihood of having EW (OR 1.58; 95%CI 1.08-2.29; p<0.05). Younger age group (11 to 14 years) was directly associated with higher likelihood of EW (OR 6.07; 95%CI 4.05-9.11; p<0.05). Adolescents whose mothers had higher education had 75% more likelihood of having AO (OR 1.75; 95%CI 1.01-3.00; p<0.05). Higher likelihood for EW and AO (OR 1.84; 95%CI 1.01-3.34; p<0.05) was identified in younger adolescents (11 to 14 years).

**Conclusions:**

Boys and younger age (11 to 14 years) were associated with a higher likelihood of EW. Adolescents whose mothers studied nine years or more were more likely to have AO. The younger age group (11 to 14 years) was associated with greater chances for the simultaneous presence of EW and AO.

## RESUMO

**Objetivo:**

Analisar os fatores sociodemográficos e do estilo de vida associados ao excesso de peso (EP), à obesidade abdominal (OA) e à presença simultânea de EP e OA em adolescentes do Sul do Brasil.

**Métodos:**

Estudo transversal de base escolar realizado com 583 adolescentes (11 a 17 anos) da cidade de Criciúma, Santa Catarina. O EP foi avaliado pelo índice de massa corpórea (IMC) e a OA, mediante perímetro da cintura (PC). As variáveis independentes analisadas foram sexo, idade, escolaridade materna, dieta balanceada, atividade física, uso de cigarro, uso de álcool em excesso e tempo de tela. Utilizou-se regressão logística binária para estimar as razões de chances (RC) e intervalos de confiança de 95% (IC95%).

**Resultados:**

Os meninos tiveram 58% de chances a mais de terem EP (RC 1,58; IC95% 1,08-2,29; p<0,05). Menor faixa etária (11 a 14 anos) foi diretamente associada a maiores chances de EP (RC 6,07; IC95% 4,05-9,11; p<0,05). Adolescentes cujas mães tinham maior escolaridade apresentaram 75% de chances a mais de terem OA (RC 1,75; IC95% 1,01-3,00; p<0,05). Maiores chances para a simultaneidade EP e OA (RC 1,84; IC95% 1,01-3,34; p<0,05) foram identificadas nos adolescentes de menor faixa etária (11 a 14 anos).

**Conclusões:**

Meninos e menor faixa etária (11 a 14 anos) estiveram associados a maiores chances para EP. Os adolescentes cujas mães estudaram nove anos ou mais apresentaram maiores chances de terem OA. Menor faixa etária (11 a 14 anos) esteve associada a maiores chances para a presença simultânea de EP e OA.

## INTRODUCTION

Overweight and obesity in children and adolescents are among major public health problems worldwide.^1^ In Brazil, research conducted in 2013 and 2014 with a representative sample of students found that 17.1% of adolescents aged 12 to 17 years were overweight, and 8.4% were obese.^2^

In adolescents, high levels of excess weight (EW) were directly associated with dyslipidemia, glucose intolerance, and hypertension.^3^ Similarly, abdominal obesity (AO) in adolescents was directly related to metabolic syndrome and systemic inflammation, a precursor of atherosclerosis.^4^ The literature has extensively described EW or AO separately.^2^ ^,^ ^5^ ^,^ ^6^ ^,^ ^7^ However, information about the simultaneous presence of these factors (EW and AO) are rarer.^1^ Health risk factors have a synergistic effect when they affect the same individual simultaneously, that is, the risk for future diseases increases in comparison to the sum of the effects of each risk factor alone.^1^

The literature has studied individual aspects, such as sociodemographic ones, usually identifying that males,^5^ ^,^ ^6^ increasing age among adolescents,^5^ and higher maternal schooling^8^ are associated with greater chances of the isolated presence of EW and AO. Characteristics related to adolescent lifestyle were directly associated with the separate presence of EW and AO, including greater consumption of unhealthy foods,^9^ low levels of physical activity,^9^ alcohol consumption,^7^ smoking, and excessive screen time.^9^ Nonetheless, little is known about the relationship between these factors and the simultaneity of EW and AO in adolescents,^1^ considering that such information could contribute to the development of better-targeted interventions to reduce obesity-related diseases.

Thus, this study aimed to investigate sociodemographic (gender, age group, and maternal schooling) and lifestyle (practice of physical activity, balanced diet, cigarette smoking, excessive alcohol consumption, and screen time-based sedentary behavior) factors associated with the isolated and simultaneous presence of EW and AO in adolescents (aged 11 to 17 years) from a city in Southern Brazil.

## METHOD

This is a school-based epidemiological study with a cross-sectional design conducted in the city of Criciúma, Santa Catarina, Brazil, in 2016. The municipality has a human development index (HDI) of 0.788, considered high, and life expectancy at birth of 75.8 years.^10^

The Human Research Ethics Committee of the Universidade do Extremo Sul Catarinense (REC/Unesc) approved this study under report No. 1,125,725 on June 26, 2015. The present investigation is part of the research “Association between health status, risk behaviors, and level of physical activity in adolescents from public schools in the city of Criciúma - Santa Catarina.” The participants signed the agreement form, and their parents/guardians, the informed consent form (ICF), authorizing the involvement of their children in the study. The questionnaires were administered in the classroom. The team of evaluators participated in previous training to standardize data collection procedures.

The target population of this study comprised 17,000 adolescents from public and private schools, enrolled from the 5^th^ grade of elementary school to the 3^rd^ grade of high school in the city of Criciúma, Santa Catarina, Brazil. The sample size calculation of the macroproject considered EW, low levels of physical activity, and low levels of aerobic fitness as the main outcomes. Taking into account previous publications conducted in the city investigated,^11^ ^,^ ^12^ the estimated prevalence for these outcomes was 30% (EW) or 70% (low levels of physical activity and aerobic fitness). The confidence level adopted was 95%, the estimated error was 5%, the design effect was 1.5, and the increment for potential losses and refusals was 20%. Given these parameters, the estimated sample consisted of 570 adolescents. A total of 583 adolescents (a representative sample of students from public and private schools in the municipality) had their data collected.

The student selection involved a two-stage cluster sampling: school (according to the type of school administration - local, state, and private) was the sampling unit of the first stage and classroom of the second stage. All institutions with elementary (from the 5^th^ grade) and high school were eligible for inclusion in the study. In the first stage, we adopted the school administration as a stratifying criterion, according to grade. In this scenario, more schools were proportionally drawn from the administration with the larger number of grades. The second stage considered the classroom density in the selected schools as a criterion to draw those where the research would be performed. All students from the selected classrooms were invited to participate in the study.

The research included adolescents aged 11 to 17 years. Prior to data collection, the exclusion criteria were:

- Health problems that prevented the performance of physical tests, such as students with special needs (blindness and physical disability, for example). The selected schools did not have cases of this type (or, at least, they were not reported by principals and/or adolescents).
- Pregnant adolescents or those who gave birth in the six months prior to data collection.

**Figure 1:**
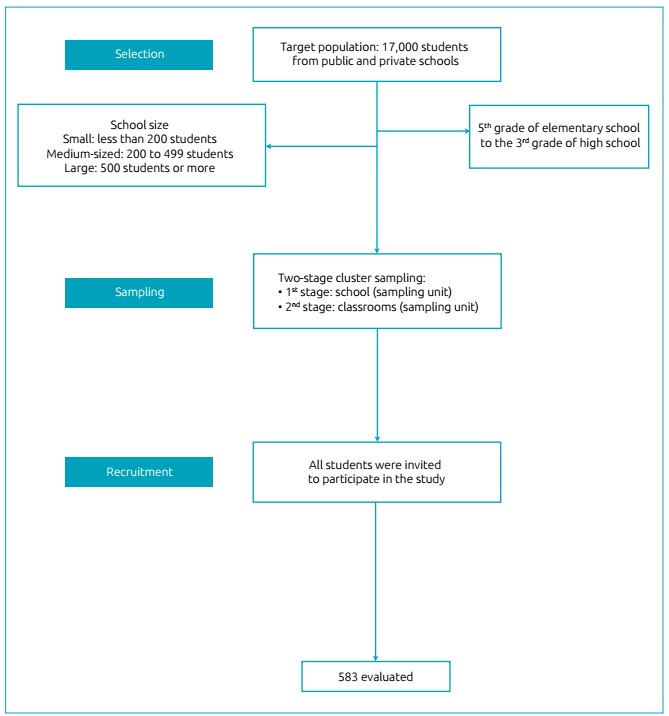
Flowchart of the study sampling process.

Adolescents who refused to participate, and those who did not have the ICF signed by their parents/guardians were not included in the study (Figure 1).

Body mass index (BMI) and waist circumference (WC) were the measurements used to classify EW and AO, respectively. Height was measured by a Sanny^®^ (São Paulo, Brazil) stadiometer with a tripod, and body mass by a G-tech^®^ (Zhongshan, China) digital scale. Weight status was classified based on the cut-off points in z-scores proposed by the World Health Organization (WHO),^13^ which defines overweight as >+1 standard deviation and obesity as >+2 standard deviations. The present study considered students with >+1 standard deviation as having EW, and those below this classification, as having “normal weight.”

WC was measured in the horizontal plane, at the midpoint between the inferior margin of the last rib and the upper border of the iliac crest,^14^ ^,^ ^15^ with a Sanny^®^ (São Paulo, Brazil) anthropometric tape. We used the cut-off points proposed previously for children and adolescents, which defined values ≥90^th^ percentile as AO, according to gender and age.^14^ ^,^ ^15^

In addition to the indicators described above, we evaluated the simultaneous presence of EW and AO in the individual. Adolescents who showed both conditions were classified as having simultaneous EW and AO.

Gender was categorized into “male” and “female”; age was collected in full years and, subsequently, dichotomized into “11 to 14” and “15 to 17” years; and maternal schooling was reported in complete years and classified as “up to eight years of study” and “nine or more years of study.”

The frequency of a balanced diet was evaluated by a questionnaire validated for Brazil.^16^ The item presented the question “Do I eat a balanced diet?” and had the following possible answers: 1) almost never; 2) rarely; 3) sometimes; 4) relatively often; and 5) almost always. We considered that the participants “often” ate a balanced diet when they responded with options 4 or 5, and “sporadically” when they selected the other options (1, 2, and 3).

Physical activity was assessed by a question from the Brazilian version of the Youth Risk Behavior Surveillance System (YRBSS) questionnaire, used in the United States and validated for the Brazilian population.^17^The question that evaluated the overall physical activity was: “During the past seven days, on how many days were you physically active for at least 60 minutes per day? (considering physical activity of moderate and/or vigorous intensity).” The answers were categorized into “little active” (zero to six days) and “active” (seven days), since the literature has reported that physical activity of moderate or vigorous intensity practiced seven days a week, for at least 60 minutes, brings benefits to the health of adolescents.^18^

Data on cigarette smoking was collected with the question^16^ “Do you smoke cigarettes?”. Individuals who declared never having smoked were considered healthy, and those who answered that they smoked more than 10 cigarettes per day; between one and ten cigarettes per day; no cigarettes in the prior six months; and no cigarettes for the previous year were included in the risk group.

Excessive alcohol consumption was evaluated based on the following question from the YRBSS questionnaire:^17^ “During the past 30 days, on how many days did you have five or more drinks of alcohol in a row?”. Those who answered that they drank this amount of alcohol at least once in the time interval mentioned were placed in the risk group for alcohol consumption.^19^

Screen time-based sedentary behavior was investigated using a questionnaire validated for Brazilian adolescents.^20^ The definition of total screen time - television, computer, and video games - followed recommendations from the literature.^21^ These variables were subsequently categorized into less than four hours/day of screen time and four or more hours/day of screen time.^21^

We used descriptive (absolute and relative frequencies) and inferential (chi-square test with Rao-Scott correction) statistics^22^. Next, we constructed binary logistic regression models, estimating odds ratios (OR) and 95% confidence intervals (95%CI). Interactions between all independent variables were tested, considering p<0.10.

The adjusted analysis of EW, AO, or the simultaneous presence of EW and AO with the other independent variables set all variables at the same level, regardless of the p-value found in the crude analysis, and those with p≤0.20 remained in the model, according to the backward method. We estimated the pseudo R^2^, the likelihood ratio, the Akaike information criterion (AIC), and the Bayesian information criterion (BIC), which indicated that the final models - assessing the relationship of EW, AO, and the simultaneous presence of EW and AO with the other independent variables - were adjusted.

The significance of the variables included in the models was verified by the Wald test, with p<0.05 indicating association. We used the Stata^®^ software (StataCorp, College Station, Texas, United States), version 13.0, for data analysis. The researcher responsible for the statistical data analysis did not participate in the collection of information.

## RESULTS

The present study involved the participation of 583 adolescents. All individuals investigated in the research had information related to the dependent variables (EW - BMI; AO - WC) gathered, and no losses were recorded. The prevalence of EW in adolescents was 33.6%; AO, 11.7%; and simultaneous EW and AO, 10.3%. Table 1 presents these prevalence rates according to the independent variables.

**Table 1:** Sample distribution according to excess weight, abdominal obesity, and their simultaneous presence in adolescent students from Criciúma, Santa Catarina, Brazil, 2016.

Variables	n (%)	Excess weight^b^			Abdominal obesity^b^			Excess weight and abdominal obesity^b^		
		n	%(95%CI)	p-value	n	%(95%CI)	p-value	n	%(95%CI)	p-value
Total	583 (100.0)	196	33.6 (29.9-37.5)		68	11.7 (9.3-14.6)		59	10.3 (7.9-12.8)	
Gender										
Male	283 (48.5)	109	55.6 (48.5-62.5)	0.01^a^	27	39.7 (28.6-52.0)	<0.12	27	45.8 (33.2-58.8)	0.64
Female	300 (51.5)	87	44.4 (37.5-51.5)		41	60.3 (48.0-71.4)		32	54.2 (41.1-66.8)	
Age group (years)										
11 to 14	308 (52.8)	156	79.6 (73.3-84.7)	0.01^a^	36	52.9 (40.8-64.7)	0.98	36	61.0 (57.7-72.8)	0.01
15 to 17	275 (47.2)	49	20.4 (15.3-26.7)		32	47.1 (35.3-59.2)		23	39.0 (27.1-47.3)	
Maternal schooling										
≤8 years	258 (44.2)	100	51.0 (43.9-58.0)	0.02^a^	22	32.3 (22.1-44.6)	0.03^a^	21	35.6 (24.2-48.9)	0.15
9 or more years	325 (55.8)	96	49.0 (42.0-56.0)		46	67.7 (55.4-77.9)		38	64.4 (51.1-75.8)	
Balanced diet										
Often	279 (47.8)	94	47.9 (40.9-55.1)	0.97	29	42.6 (31.2-54.9)	0.35	24	40.7 (28.6-53.9)	0.24
Sporadically	304 (52.2)	102	52.0 (45.0-59.0)		39	57.4 (45.1-68.8)		35	59.3 (46.0-71.3)	
Physical activity										
Active	26 (4.5)	12	6.1 (3.5-10.5)	0.17	02	3.0 (0.7-11.3)	0.51	02	3.3 (0.8-13.0)	0.67
Little active	557 (95.5)	184	93.9 (89.4-96.5)		66	97.0 (88.6-99.3)		57	96.7 (86.7-99.2)	
Cigarette smoking										
No	533 (91.4)	177	90.3 (85.2-93.7)	0.49	65	95.6 (86.8-98.6)	0.19	56	94.9 (84.9-98.4)	0.31
Yes	50 (8.6)	19	9.7 (6.2-14.7)		03	4.4 (1.4-13.1)		03	5.1 (1.6-15.0)	
Excessive alcohol consumption										
No	459 (78.7)	162	82.6 (76.6-87.4)	0.10	50	73.5 (61.5-82.8)	0.28	45	76.3 (63.4-85.6)	0.63
Yes	124 (21.3)	34	17.4 (12.6-23.3)		18	26.5 (17.1-38.5)		14	23.7 (14.4-36.6)	
Screen time										
<4 hours/day	211 (36.2)	70	35.7 (29.2-42.7)	0.86	30	44.1 (32.6-56.3)	0.15	27	45.8 (33.2-58.8)	0.11
≥4 hours/day	372 (63.8)	126	64.3 (57.3-70.7)		38	55.9 (43.6-67.4)		32	54.2 (41.1-66.8)	

n: sample size; 95%CI: 95% confidence interval; ^a^p<0.05; ^b^Pearson’s chi-square test for the difference between excess weight, abdominal obesity, and simultaneous excess weight and abdominal obesity with the covariates.

In the adjusted analysis between EW and the other independent variables, male adolescents and those in the age group 11 to 14 years were, respectively, 1.58 (95%CI 1.08-2.29) and 6.07 (95%CI 4.05-9.11) more likely to have EW. The final model of the associations tested comprised the variables gender and age group, which were able to explain 12.8% (pseudo R^2^=0.1278) of the EW variation (Table 2).

**Table 2:** Odds ratios and 95% confidence intervals in the association between excess weight, abdominal obesity, and independent variables in adolescent students from Criciúma, Santa Catarina, Brazil, 2016.

Variables	Excess weight				Abdominal obesity			
	Crude analysis		Adjusted analysis^b^		Crude analysis		Adjusted analysis^b^	
	OR	(95%CI)	OR	(95%CI)	OR	(95%CI)	OR	**(95%CI)**
Gender								
Female	1	(1.08-2.17)	1	(1.08-2.29)	1	(0.39-1.11)	1	(0.41-1.18)
Male	1.53		1.58^a^		0.66		0.70	
Age group (years)								
15 to 17	1	(4.03-9.02)	1	(4.05-9.11)	1	(0.60-1.66)	1	(0.75-2.27)
11 to 14	6.03		6.07^a^		0.99		1.31	
Maternal schooling								
≤8 years	1	(0.46-0.93)	1	(0.58-1.24)	1	(1.03-3.03)	1	(1.01-3.00)
9 or more years	0.66		0.85		1.77^a^		1.75	
Balanced diet								
Often	1	(0.70-1.40)	1	(0.77-1.64)	1	(0.57-3.85)	1	(0.73-2.06)
Sporadically	0.99		1.12		1.48		1.23	
Physical activity								
Active	1	(0.26-1.27)	1	(0.25-1.42)	1	(0.37-7.02)	1	(0.30-5.91)
Little active	0.57		0.59		1.62		1.33	
Cigarette smoking								
No	1	(0.68-2.24)	1	(0.47-1.83)	1	(0.13-1.50)	1	(0.82-2.74)
Yes	1.23		0.93		0.45		1.50	
Excessive alcohol consumption								
No	1	(0.44-1.07)	1	(0.71-1.95)	1	(0.77-2.46)	1	(0.82-2.74)
Yes	0.69		1.18		1.38		1.50	
Screen time								
<4 hours/day	1	(0.72-1.47)	1	(0.59-1.30)	1	(0.41-1.14)	1	(0.40-1.15)
≥4 hours/day	1.03		0.87		0.68		0.68	

OR: odds ratio; 95%CI: 95% confidence interval; ^a^p<0.05, adjusted for all covariates; ^b^analysis according to other covariates, keeping those with p≤0.20. For the association between excess weight and other covariates, the final model comprising the variables gender and age group showed a pseudo R^2^=0.1292, Akaike information criterion (AIC)=674.48, and Bayesian information criterion (BIC)=691.95. Compared to the saturated (pseudo R^2^=0.1344, AIC=680.52, and BIC=719.84) and null (pseudo R^2^=0, AIC=767.40, and BIC=771.77) models, the final model had a value of 0.55 and <0.001, respectively, according to the likelihood ratio test. For the association between abdominal obesity and other covariates, the final model comprising the variables gender, maternal schooling, alcohol consumption, cigarette smoking, and screen time-based sedentary behavior showed a pseudo R^2^=0.0297, AIC=418.77, and BIC=444.95. Compared to the saturated (pseudo R^2^=0.0350, AIC=422.52, and BIC=461.79) and null (pseudo R^2^=0, AIC=421.21, and BIC=425.57) models, the final model had a value of 0.52 and 0.03, respectively, according to the likelihood ratio test.

Adolescents whose mothers had higher schooling (nine or more years) presented 75% more chances (95%CI 1.01-3.00) of having AO. The final model of the dependent variable AO included the variables gender, maternal schooling, alcohol consumption, cigarette smoking, and screen time-based sedentary behavior, which were able to explain approximately 3.0% (pseudo R^2^=0.0297) of the AO variation (Table 2).

Adolescents in the age group 11 to 14 years were 84% more likely (95%CI 1.01-3.34) to having both EW and AO. The final model of the associations tested comprised the variables age group, maternal schooling, alcohol consumption, cigarette smoking, and screen time-based sedentary behavior, which were able to explain 2.7% (pseudo R^2^=0.0277) of the variation in simultaneous EW and AO. This (final) model presented values close to those from the saturated model and better than the ones from the null model, indicating that the variables included were adjusted for the outcome and among themselves (Table 3).

**Table 3:** Odds ratios and 95% confidence intervals in the association of the simultaneous presence of excess weight and abdominal obesity with independent variables among adolescents from public schools in Criciúma, Santa Catarina, Brazil, 2016.

Variables	OR	Crude analysis	p	OR	Adjusted analysis^b^	
		(95%CI)			(95%CI)	p
Gender						
Female	1	(0.51-1.51)	0.64	1	(0.53-1.60)	0.77
Male	0.88			0.92		
Age group (years)						
15 to 17	1	(0.83-2.52)	0.05	1	(1.01-3.34)	0.04
11 to 14	1.45			1.84^a^		
Maternal schooling						
≤8 years	1	(0.85-2.62)	0.16	1	(0.92-2.89)	0.09
9 or more years	1.49			1.63		
Balanced diet						
Often	1	(0.80-2.40)	0.24	1	(0.79-2.40)	0.26
Sporadically	1.39			1.37		
Physical activity						
Active	1	(0.31-5.95)	0.67	1	(0.27-5.28)	0.82
Little active	1.37			1.19		
Cigarette smoking						
No	1	(0.16-1.80)	0.24	1	(0.12-1.49)	0.18
Yes	0.54			0.43		
Excessive alcohol consumption						
No	1	(0.61-2.20)	0.63	1	(0.79-3.20)	0.19
Yes	1.16			1.59		
Screen time						
<4 hours/day	1	(0.37-1.10)	0.82	1	(0.34-1.03)	0.07
≥4 hours/day	0.64			0.59		

OR: odds ratio; 95%CI: 95% confidence interval; ^a^p<0.05, adjusted for all covariates; ^b^analysis according to other covariates, removing those with p≤0.20. The final model comprising the variables age group, maternal schooling, alcohol consumption, cigarette smoking, and screen time-based sedentary behavior showed a pseudo R^2^=0.0277, Akaike information criterion (AIC)=383.33, and Bayesian information criterion (BIC)=409.53. Compared to the saturated (pseudo R^2^=0.0335, AIC=387.12, and BIC=426.41) and null (pseudo R^2^=0, AIC=383.90, and BIC=388.26) models, the final model had a value of 0.87 and <0.001, respectively, according to the likelihood ratio test.

## DISCUSSION

The results obtained in the present study regarding the high prevalence of EW among adolescents (33.6%) agree with findings from the National Adolescent Student Health Survey (*Pesquisa Nacional de Saúde do Escolar* - PeNSE), which estimated the prevalence of adolescents with EW as 31.5% in 2015.^23^Although the etiology of obesity is multifactorial, involving both environmental and genetic factors in its genesis, changes in dietary and physical activity patterns are the determinants that most contribute to the rise in EW.^24^ Therefore, the high prevalence of EW identified in this study might be associated with the constant urbanization process characteristic of emerging countries like Brazil, which has helped to increase barriers to the implementation of actions directly related to the maintenance of and/or reduction in body weight, such as physical activity, either by difficulties in accessing places conducive to the practice of such activities or even by the lack of these spaces.^25^

The prevalence of AO (11.7%) found in the present study was similar to those reported in investigations carried out in other Brazilian cities.^26^ ^,^ ^27^ ^,^ ^28^ Results from different studies should be compared and analyzed with caution, given the distinct cut-off points they adopt to classify AO.^26^ ^,^ ^27^ ^,^ ^28^ The proposal of accurate WC cut-off points to diagnose AO is based on high sensitivity and specificity values to identify body fat in the population assessed.^26^This research and a study conducted in the city of Curitiba, Paraná, adopted a cut-off point based on WC percentiles to classify AO.^5^ Investigations carried out in the cities of São José (Santa Catarina)^26^ and Londrina (Paraná)^28^ used cut-off points indicated by dual-energy absorptiometry x-ray to diagnose AO.^29^Despite the lack of consensus concerning the reference standard to classify adolescents with AO,^15^ the use of percentiles have been encouraged, given their ability to compensate for different developmental stages and ethnicity in the young population.^15^

The estimated prevalence of adolescents who presented EW and AO simultaneously in the present study was 10.3%. Even though the literature on the simultaneous presence of EW and AO in adolescents is scarce,^1^ health issues, such as cardiometabolic diseases, dyslipidemia, decreased glucose tolerance, reduced insulin sensitivity, and early mortality, were directly associated with both conditions.^30^ Also, the accumulation of risk factors in an individual might be more strongly related to adverse health conditions when compared to the presence of each factor alone, which reinforces the relevance of our findings.^3^ ^,^ ^4^ Thus, the proposal of actions aimed at preventing the emergence of these diseases becomes necessary, including the implementation of health education programs addressing obesity-related problems in the school environment targeted at adolescents, parents, and guardians, given the multicausality of this condition.

The findings of the present study revealed that boys had 58% more chances of having EW than girls. These data agree with results found in national literature.^6^ A hypothesis that could justify this finding suggests that girls are more dissatisfied with their body weight and under greater social pressure to maintain a standard of thinness.^31^ Besides, during adolescence, girls tend to be more concerned about being perceived as attractive by their peers, which could contribute to the maintenance of body weight.^31^ In addition to the justifications mentioned above, puberty has been considered a crucial period for the development of EW.^32^ In the present study, approximately one in every four male students assessed belonged to the age group 11 to 14 years (data not shown in tables), and part of these individuals might have been in early maturation stages, which are directly related to greater accumulation of body fat and, consequently, to EW.^32^

Adolescents whose mothers had a higher level of schooling (nine or more years) were more likely to have AO, a result similar to those identified in the literature.^33^ ^,^ ^34^ Although higher maternal schooling is directly associated with the qualitative aspects of their children’s food intake, the concern of mothers with the nutritional status of their children is more closely related to the quantity rather than the quality of the food consumed, which could lead to AO.^34^ Moreover, with their increased level of education and participation in the labor market, mothers have less time for family care, facilitating the influence of advertising and third parties in imposing inadequate eating habits, resulting in AO in their children.^34^

In the present study, adolescents aged 11 to 14 years had greater chances of having EW and presenting both EW and AO when compared to those aged 15 to 17 years. A possible explanation could be related to the fact that this research identified a higher prevalence of adolescents physically inactive (n=280; 53.5%) and who would normally spend more time on screen time-based sedentary behavior (n=205; 55.1%) in the age group 11 to 14 years when compared to those aged 15 to 17 years (data not presented in tables/figures). Such conditions (physical inactivity and screen time-based sedentary behavior) are directly associated with lower energy expenditure, which can contribute to EW and the simultaneous presence of EW and AO.

The lack of assessment of sexual maturation, an aspect directly related to EW and AO in adolescents, is a limitation of the present study. Other limitations include the cross-sectional design, preventing the establishment of causality and temporality between the indicators of body composition and other variables, as well as the collection of information with a self-administered questionnaire, which can result in response bias from the individuals investigated. Furthermore, despite the present study having selected the students by cluster sampling, the classroom density did not take into account the school hours, leading to a lack of representativeness of students from the periods evaluated (daytime or nighttime), considered another limitation of the research*.* However, the identification of subgroups of adolescents more prone to developing risk factors for adverse health conditions is a strength of the study.

We can conclude that approximately one in every three adolescents had EW. Out of all participants, 11.7% had AO, and 10.3% presented both AO and EW. Male adolescents and those from a younger age group (11 to 14 years) showed greater chances of having EW. Adolescents whose mothers had nine or more years of study were more likely to have AO. Also, the younger age group (11 to 14 years) was associated with a higher probability of simultaneously presenting EW and AO.
